# Cavity Shaving plus Lumpectomy versus Lumpectomy Alone for Patients with Breast Cancer Undergoing Breast-Conserving Surgery: A Systematic Review and Meta-Analysis

**DOI:** 10.1371/journal.pone.0168705

**Published:** 2017-01-03

**Authors:** Ke Wang, Yu Ren, Jianjun He

**Affiliations:** Department of Breast Surgery, the First Affiliated Hospital of Xi’an Jiaotong University, Xi’an, Shaanxi Province, China; Tata Memorial Centre, INDIA

## Abstract

The margin status is a well-established prognostic predictor for patients undergoing breast-conserving surgery (BCS). Recent data suggested that cavity shaving in addition to lumpectomy might be a promising approach for improving the clinical outcomes. We aimed to compare the efficacy and safety between cavity shaving plus lumpectomy and lumpectomy alone with a systematic review and meta-analysis. We searched the PubMed, Embase, and Cochrane CENTRAL databases for studies comparing cavity shaving with lumpectomy before June 10, 2016. Both comparative studies and self-control studies were included. A random-effects model was used to estimate the odds ratios (ORs) for positive margin rate, reoperation rate, recurrence rate, and weighted mean difference (WMD) for excised tissue volume. Twenty-six studies were included in the meta-analysis. The cavity shaving group had a significantly lower positive margin rate than the BCS-alone group (16.4% vs. 31.9%; OR = 0.41, 95% CI 0.32–0.53, P < 0.05). Cavity shaving was associated with a significantly decreased rate of reoperation (OR = 0.42, 95% CI 0.30–0.59, P < 0.05). The overall locoregional rate was low for cavity shaving and BCS-alone (3% vs. 4%). Cavity shaving had no significant effect on the risk of locoregional recurrence (OR = 0.86, 95% CI 0.32–2.35; P = 0.78). The excised tissue volume did not differ substantially between cavity shaving and BCS alone (WMD = −23.88, 95% CI −55.20 to 7.44, P = 0.14). For patients undergoing BCS, additional cavity shaving was an effective method to decrease the positive margin rate and avoid reoperation. The addition of cavity shaving did not appear to have excessive excised tissue volume compared with partial mastectomy alone.

## Introduction

Breast cancer is the most common malignancy among women in the United States [1]. Nearly 270,000 women were diagnosed with operable breast cancer in 2015, approximately two-thirds (180,000 women) of whom were suitable for breast-conserving surgery (BCS), namely partial mastectomy [2]. For early cases, BCS can yield an equivalent survival compared with radical mastectomy [3,4]. However, BCS has a higher lifelong local recurrence rate than total mastectomy, mandating adjuvant radiation therapy [5], and approximately 20–35% of patients who undergo BCS eventually require reoperation [6,7]. Margin status is a pivotal predictor for local recurrence [5,8,9]. The rate of positive margins after a partial mastectomy is as high as 20–40%. Patients with breast cancer with positive margins have a two-fold increase in the risk of tumor recurrence compared with those who have negative margins [10].

Cavity shaving (CS) was first introduced as a pathological biopsy technique to examine the residual tumor during or after partial mastectomy, and the incidence of residual tumor bed positivity reaches as high as 39.3% [11–13]. Later, several studies demonstrated that CS could be an easy and effective procedure to decrease the positive margin rate and re-excision rate. However, some authors have argued that the excision of selective margins might be sufficient [14,15]. The value of CS has been questioned because adjacent multifocal disease might outweigh margin status in causing BCS failure [16]. Thus, we conducted this systematic review and meta-analysis with the aim to compare the efficacy and safety between CS plus lumpectomy and lumpectomy alone.

## Materials and Methods

We searched the PubMed, Embase, and Cochrane CENTRAL databases for eligible studies published before June 10, 2016. The following groups of key words or medical terms were used: (“cavity shave” or “cavity shaving” or “cavity margin” or “shave margin”) and (“lumpectomy” or “breast-conserving” or “partial mastectomy” or “breast cancer”). The detailed search strategy used for PubMed is listed in S1 File. The language was limited to English. Additionally, the reference lists of relevant studies were searched for potentially eligible records. This systematic review and meta-analysis was performed following the PRISMA guideline (S2 File) [17].

### Study inclusion

The included studies could be comparative studies, including randomized controlled trials (RCTs) or non-randomized studies (NRS), or self-control studies, which compared CS with standard lumpectomy in patients with breast cancer undergoing breast-conserving surgeries. The breast cancer stage ranged from 0 to Ⅲ. We preferred the margin assessed by the “no ink on tumor” criteria [10]. Other wider margins criteria were also accepted. However, studies assessing margins by imaging-guide techniques were excluded. We only selected studies that applied the CS procedure at the initial surgery. When multiple groups were presented in an individual studies, we selected the comparison between BCS plus CS and BCS alone. The outcomes of interest included positive margin rate, reoperation rate, locoregional or distant recurrence rate, volume of excised tissues, and cosmetic outcomes.

### Data collection and quality assessment

Two reviewers independently extracted the data using a standardized form. When an outcome was followed at different intervals, the one with longest follow-up was selected. The following information was extracted: author, year, study design, country, sample size, age, proportion of ductal carcinoma in situ (DCIS), tumor size, outcomes of interest, and study period. To assess the quality of the RCTs, we used the Jadad score [18]. We used the modified Newcastle-Ottawa scale (NOS) to evaluate non-randomized or non-comparative studies [19]. The NOS evaluated four aspects including selection, comparability, exposure, and outcomes. The scores ranged from 0–7 points, with 0–2 points indicating low quality, 3–5 points indicating medium quality, and ˃6 points denoting high quality.

### Statistical analysis

The meta-analysis for comparative outcomes was performed using Stata 12.0 software (Stata Corporation, College Station, TX, USA). For pooling the single-arm event rate, we used Comprehensive Meta-Analysis software (version 2.2, Biostat, Englewood, NJ, USA). For dichotomous outcomes, we used odds ratios (ORs) and 95% confidence intervals (CI) as the effect estimate. The relative risk (RR) was only used in sensitivity analysis for pooling data from RCTs. For continuous measures, we used the weighted mean difference (WMD) with 95% CI as the effect estimate. The median value with range or interquartile range was transformed into the mean ± standard deviation (SD) by the established calculation method [20]. A random-effects model was used for data synthesis. Heterogeneity was assessed by the I^2^ statistics. I^2^ <25% was regarded as low heterogeneity, 25–75% was regarded as medium heterogeneity, and ≥75% was considered high heterogeneity [21]. Subgroup analysis was performed by the study design and region. Sensitivity analysis was performed by excluding the included studies one by one. Meta-regression was conducted according to the sample size and percentage of DCIS. The publication bias was examined by the funnel plot, and quantitatively by the Egger’s test and Begg’s test [22,23]. Two-sided P < 0.05 was considered statistical significant.

## Results

### Selection process

**Fig 1:**
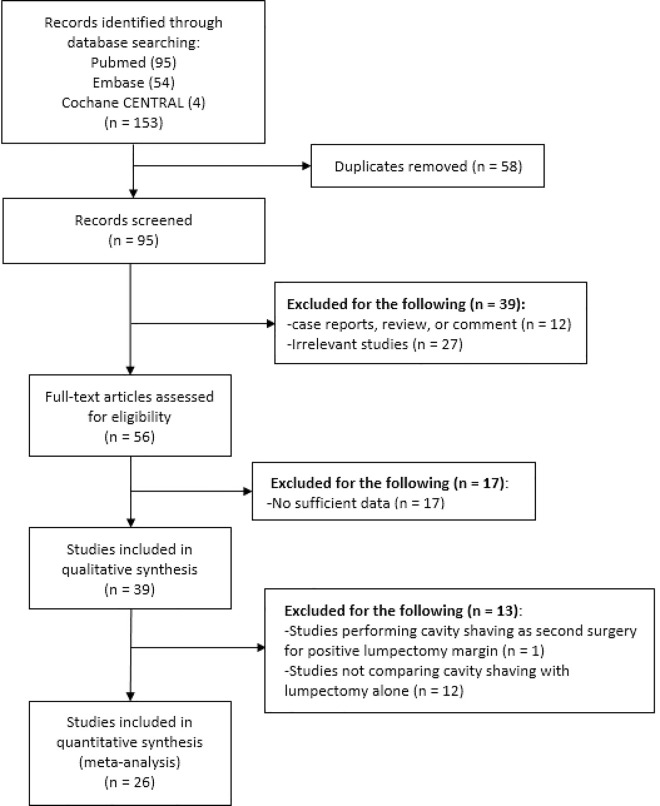
Flow Diagram Showing the Study Selection Process.

A total of 153 records were initially identified, including 95 records from the PubMed, 54 records from the Embase, and 4 records from the Cochrane CENTRAL databases. After removing duplicates and irrelevant publications, 56 full-text studies were assessed for eligibility. Thirty-nine studies were pooled into a qualitative synthesis. Further, we excluded 12 studies that did not compare CS with BCS alone, and 1 study in which CS was performed as a second surgery. Twenty-six studies were included into the meta-analysis. Fig 1 displays the study selection process.

### Study characteristics

Twenty-six studies between 1994 and 2016 were included into the final meta-analysis, including 15 comparative studies (2 RCTs and 13 NRSs) [15,24–37], and 11 self-control NRSs [12,16,38–46]. Among the 24 NRSs, most had a retrospective design (21/24). The characteristics of the included studies are presented in Table 1. Eleven studies were conducted in Europe, 13 in North America, and 2 in China. The sample size ranged from 76–786. The median or mean age for the majority of included studies ranged from 50–60 years. Although early patients with breast cancer indicated for BCS were unanimously included, the percentage of DCIS varied greatly (0%-100%). The mean/median size of tumor ranged from 1–2.5 cm. In quality assessment, 2 RCTs achieved high-quality scores [25,27]. When assessing the NRSs by the NOS score, the scores ranged from 5–7. The items of representativeness of cases undergoing CS and adequate follow-up were least fulfilled. The quality evaluation is displayed in S1 and S2 Tables.

**Table 1:** Characteristics of the Included Studies.

Author (year)	Country	Design	No. of patients	Age (year)	DCIS, n (%)	Treatment regimens	Median size (cm)	Outcomes	Study period
Macmillan et al. (1994)	UK	Retrospective	264	Median: 55	NA	Shave-after vs. shave-before	1.3	Positive margin rate; recurrence rate	1988–1992
Keskek et al (2004)	UK	Retrospective	301	Mean: 55	20 (6.6%)	Shave-after vs. shave-before	2.0	Positive margin rate; reoperation rate; recurrence rate	1997–2002
Camp et al. (2005)	USA	Retrospective	257	Median: 58	47 (17.6%)	Shave vs. non-shave	NA	Reoperation rate; recurrence rate	1989–2001
Cao et al. (2005)	USA	Retrospective	126	Mean: 58	23 (18.3%)	Shave-after vs. shave-before	1.4	Positive margin rate	2003–2004
Janes et al. (2006)*	UK	Prospective	111	Median: 59	1 (1%)	Shave-after vs. shave-before	1.9	Positive margin rate; reoperation rate	2001–2003
Huston et al. (2006)	USA	Retrospective	171	Median: 59	29 (17%)	Shave vs. non-shave	1.3	Positive margin rate; reoperation rate; volume of excised tissue	2000–2006
Jacobson et al. (2008)	USA	Retrospective	125	NA	26 (20.8%)	Shave-after vs. shave-before	1.8	Positive margin rate; reoperation rate	2002–2006
Marudanayagam et al. (2008)	UK	Retrospective	786	Mean: 58	0	Shave vs. non-shave	1.7	Reoperation rate	2000–2005
Povoski et al. (2009)	USA	Retrospective	204	Median: 57	0	Shave-after vs. shave-before	1.6	Positive margin rate; volume of excised tissue	2003–2007
Lovrics et al. (2009)	Canada	Retrospective	489	Mean: 59	0	Shave vs. non-shave	NA	Positive margin rate; reoperation rate	2000–2002
Tengher-Barna et al. (2009)	France	Retrospective	107	Median: 57	15 (14%)	Shave-after vs. shave-before	1.6	Positive margin rate; reoperation rate	2003–2006
Rizzo et al. (2010)	USA	Retrospective	320	Mean: 59	88 (44.2%)	Shave vs. non-shave	1.6	Positive margin rate; reoperation rate	2004–2007
Zavagno et al. (2010)	Italy	Retrospective	508	Mean: 58	0	Shave vs. non-shave	1.6	Positive margin rate; reoperation rate; volume of excised tissue	2001–2008
Coopey et al. (2011)	USA	Retrospective	773	Mean: 56	223 (28.8%)	Shave vs. non-shave	1.7	Reoperation rate; recurrence rate	2004–2006
Feron et al. (2011)	France	Prospective	96	Mean: 56	0	Shave-after vs. shave-before	1.4	Positive margin rate; reoperation rate	Jan-Dec 2007
Hequet et al. (2011)	France	Retrospective	99	Median: 58	16 (16.1%)	Shave-after vs. shave-before	1.5	Positive margin rate	2007–2008
Kobbermann et al. (2011)	USA	Retrospective	138	Median: 59	40 (29%)	Shave vs. non-shave	NA	Positive margin rate; reoperation rate	2004–2009
Wolf et al. (2011)	USA	Retrospective	356	Mean: 58	356 (100%)	Shave vs. non-shave	NA	Positive margin rate; reoperation rate; volume of excised tissue	2004–2008
Mook et al. (2012)	USA	Retrospective	144	Median: 59	42 (29.2%)	Shave vs. non-shave	1.5	Positive margin rate; reoperation rate; volume of excised tissue; complications	2004–2009
Unzeitig et al. (2012)	USA	Retrospective	522	Mean: 57	384 (73.6%)	Shave vs. non-shave	NA	Reoperation rate	NA
Yang et al. (2012)	China	Prospective	166	Median: 49	24 (14.7%)	Shave-after vs. shave-before	2.1	Positive margin rate	2008–2009
Hequet et al. (2013)	France	Retrospective	294	Median: 57	35 (12%)	Shave-after vs. shave-before	1.2	Positive margin rate; recurrence rate	2003–2008
Bolger et al. (2015)	Ireland	Retrospective	188	Mean: 54	0	Shave vs. non-shave	NA	Positive margin rate; reoperation rate	2008–2011
Chagpar et al. (2015)	USA	RCT	235	Mean: 61	56 (23.8%)	Shave vs. non-shave	1.1	Positive margin rate; reoperation rate; volume of excised tissue; complications	2011–2013
Jones et al. (2016)	USA	RCT	76	Mean: 60	13 (17.1%)	Shave vs. non-shave	2.3	Positive margin rate; recurrence rate; volume of excised tissue	2009–2012
Pata et al. (2016)	Italy	Retrospective	298	Median: 61	40 (13.4%)	Shave vs. non-shave	1.2	Positive margin rate; reoperation rate; volume of excised tissue; recurrence rate	Jan-Dec 2013

DCIS, ductal carcinoma in situ; NA, not available; RCT, randomized controlled trial.

* One group was excluded because CSM was not unanimously performed.

### Positive margin rate

**Fig 2:**
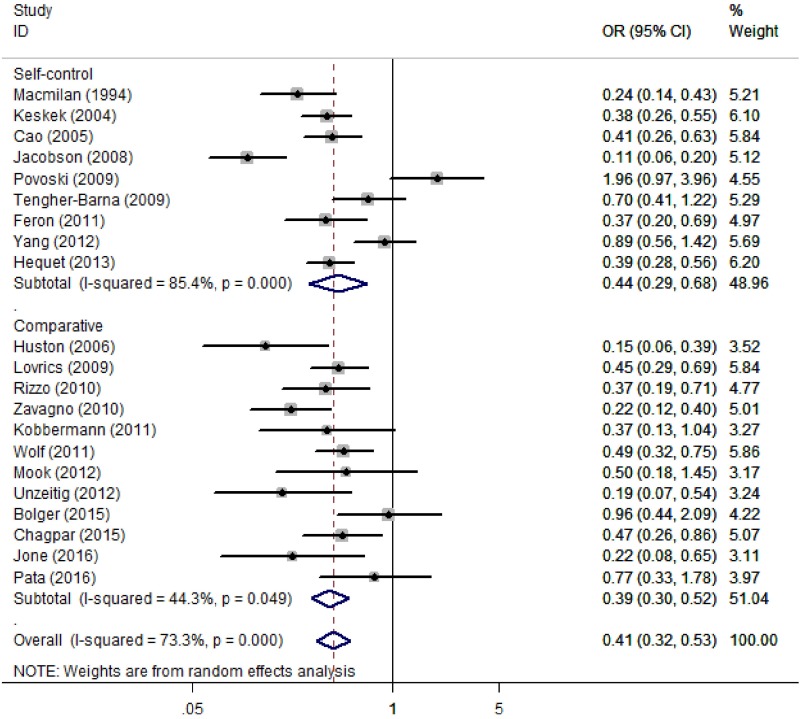
Forest Plot Comparing the Positive Margin Rate for Partial Mastectomy With and Without Cavity Shaving.

Twenty-two studies compared the positive margin rate between CS plus BCS with BCS alone. Because 2 studies by the same author had overlapping cohorts [40,41], the one with the largest sample size was selected for analysis [41]. Overall, the pooled positive margin rate for the CS procedure was 16.4% (95% CI 12.8–20.7%, I^2^ = 87.7%), and the pooled rate for BCS alone was 31.9% (95% CI 26.1–38.4%, I^2^ = 93.4%). In comparative analysis, CS plus BCS could significantly reduce the positive margin rate compared to BCS alone (OR = 0.41, 95% CI 0.32–0.53, P < 0.05). High heterogeneity was present (I^2^ = 73.3%; P < 0.05) (Fig 2). The association remained significant for self-control studies (OR = 0.44, 95% CI 0.29–0.68, P < 0.05; I^2^ = 85.4%) and comparative studies (OR = 0.39, 95% CI 0.30–0.52, P < 0.05; I^2^ = 44.3%). When stratified by region (Europe, North America, and China), only 2 Chinese studies showed a non-significant pooling result (OR = 0.60, 95% CI 0.28–1.30, P = 0.19).

**Fig 3:**
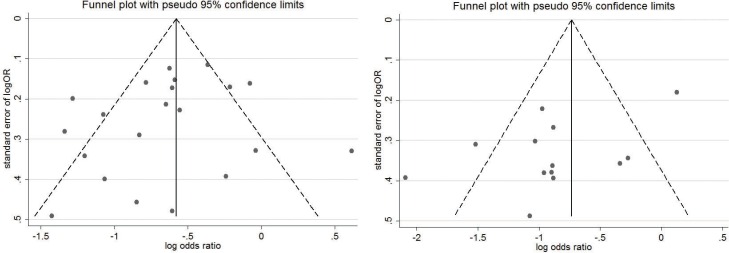
The Publication Bias Shown by Funnel Plots. (A) Funnel plot for studies comparing the positive margin rate; (B) Funnel plot for studies comparing the reoperation rate.

In the sensitivity analysis, we specifically analyzed the 2 RCTs by using the relative risk (RR) as estimates [25,27], and this analysis still showed a significant correlation (RR = 0.53, 95% CI 0.44–0.64; I^2^ = 19.2%). When excluding the included studies one by one, no individual study accounted for a significant change in the pooled data. In the meta-regression, sample size and the proportion of DCIS did not appear to account for the source of heterogeneity (P = 0.32 and P = 0.67, respectively). The funnel plot was symmetrical (Fig 3A), and no publication bias was detected by the Begg’s test (P = 0.45) or Egger’s test (P = 0.09).

### Reoperation rate

**Fig 4:**
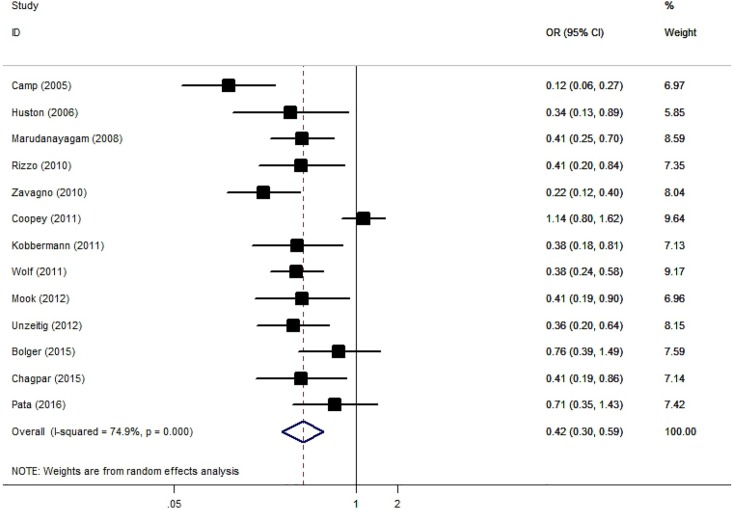
Forest Plot Comparing the Reoperation Rate for Lumpectomy With and Without Cavity Shaving.

Thirteen comparative studies were available. The pooled reoperation rate was 15.0% (95% CI 9.3–23.3%; I^2^ = 94.5%, P < 0.05) for the CS group, and was 30.1% (95% CI 21.8–39.9%; I^2^ = 95.0%, P < 0.05) for BCS alone. CS plus partial mastectomy could significantly reduce the reoperation rate when compared to standard partial mastectomy (OR = 0.42, 95% CI 0.30–0.59, P < 0.05) (Fig 4). A significantly high heterogeneity was shown (I^2^ = 74.9%, P < 0.05). A sensitivity analysis by excluding one individual record at a time did not detect a significant change for any study. In the meta-regression analysis, neither the sample size (P = 0.36) nor the proportion of DICS (P = 0.91) could explain the source of heterogeneity. The funnel plot was symmetrical (Fig 3B). No publication bias was revealed by Begg’s test (P = 0.58) or Egger’s test (P = 0.44).

### Recurrence

**Fig 5:**
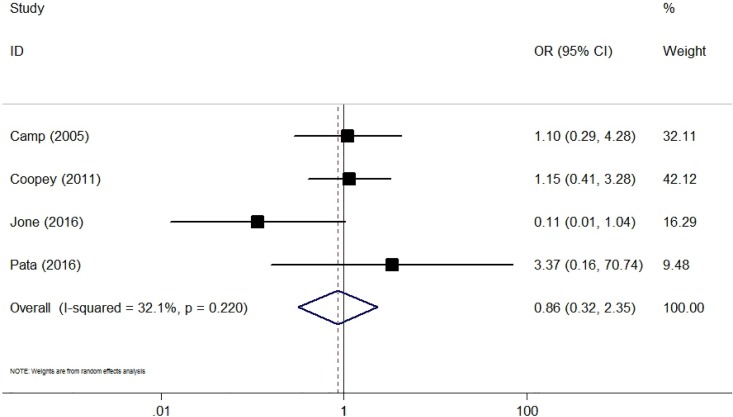
Forest Plot Comparing the Locoregional Recurrence Rate for Lumpectomy With and Without Cavity Shaving.

Four studies reported data on locoregional recurrence [15,27,32,37]. The pooled incidence of locoregional recurrence for the CS group was 3.0% (95% CI 2.0–4.5%), and for the standard partial mastectomy group was 4.0% (95% CI 1.1–13.4%). The pooled result showed that CS in addition to BCS reduced the incidence of locoregional recurrence by 14% but this was not statistically significant (OR = 0.86, 95% CI 0.32–2.35; P = 0.78) (Fig 5). The heterogeneity was low and non-significant (I^2^ = 32.1%, P = 0.22). Only 2 studies compared additional CS with BCS alone on distal recurrence [15,27]. No significant result could be detected (OR = 1.75, 95% CI 0.18–17.04, P = 0.63; I^2^ = 60.0%, P = 0.11).

### Excised tissue volume and cosmetic outcome

**Fig 6:**
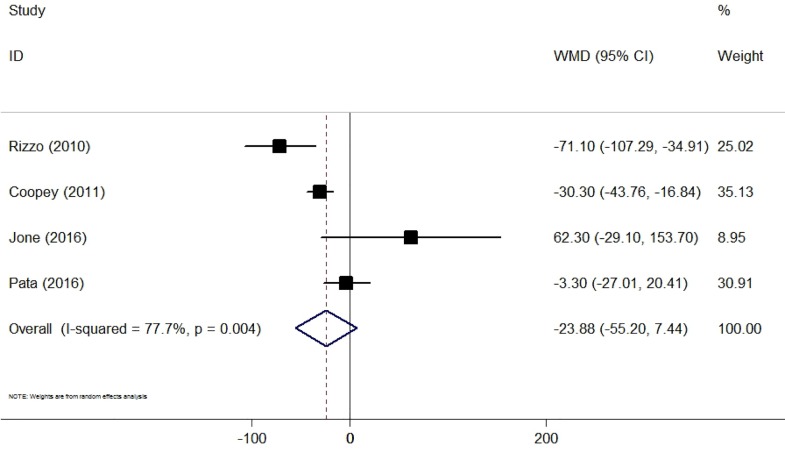
Forest Plot Comparing the Excised Tissue Volume for Lumpectomy With and Without Cavity Shaving.

Eight studies compared the excised tissue volume between CS plus BCS and BCS alone [15,25,27,31–33,35,36]. Four studies were eligible for data synthesis [15,27,32,33]. The pooled data revealed that CS did not have a significantly increased volume of excised tissue compared with BCS alone, with a high level of heterogeneity (WMD = −23.88, 95% CI −55.20 to 7.44, P = 0.14; I^2^ = 77.7%, P < 0.05) (Fig 6). Two studies reported the cosmetic outcome. Mook et al. demonstrated that patients undergoing CS had improved cosmesis compared with those undergoing partial mastectomy [31]. Chagpar et al. showed a non-significant difference between the CS group and the BCS alone group in the patients’ perception of their cosmetic outcomes [25].

## Discussion

In early years, cavity shave sampling was utilized as a pathological procedure to examine residual disease in the remnant cavity or tumor bed [13,47]. Frequently, residual tumor was detected in the resected cavity specimen [11,13,48]. A positive margin was shown to be associated with a lower reoperation and re-excision rates than a negative margin [16,43,44,48–50]. However, the cavity biopsy differed from cavity resection in extent and width [16,48]. The variability of cavity sampling techniques has been elaborately summarized before [16]. In contrast, the technique of CS is relatively consistent, with complete resection of the surface and sufficient width of the cavity wall.

Our findings suggested that additional CS had a lower positive margin rate than BCS alone (16.4% vs. 31.9%). CS was associated with a 59% OR reduction in the tumor-involved margin. The precision of this association was reinforced by the narrow 95% CI of 0.32–0.53 [51]. The statistical significance was reinforced by stratified analysis of self-control or comparative studies, and by the pooled data from 2 RCTs. To date, the NCT01452399 trial represented the most convincing evidence with regard to CS [25]. Notably, this trial demonstrated a significant superiority for cavity shaving in univariate analysis (P = 0.01) but not in multivariate analysis (P = 0.06). In this meta-analysis, we included a large number of studies with greater statistical power and proved the advantage of CS.

CS with BCS could approximately halve the reoperation rate compared with BCS alone (15.0% vs. 30.1%). In accordance with the trial by Chagpar et al. [25], this meta-analysis showed that CS was associated with a significantly reduced reoperation rate (OR = 0.42). The statistical power was also reinforced by the narrow 95% CI of 0.30–0.59. CS had a non-significant impact on the rate of locoregional recurrence. However, the pooled local recurrence rate was low (3% vs. 4%). CS did not seem to correlate with decreased distant recurrence. Recurrence is an outcome that requires long-term follow-up and might be influenced by multiple clinicopathological factors, such as genetic mutation, histology type, and neoadjuvant chemotherapy (NAC). In addition, the very small number of studies limits the statistical power.

One of the major concerns related to CS is the excessive resection of tissue volume and the subsequent poor aesthetic result. However, we did not find that CS significantly excised more tissue volume than standard partial mastectomy. In fact, the excision extent was largely at the discretion of individual surgeons, some of whom might have decided to perform wide resection to ensure a negative margin when the cavity wall was not removed. Although data on cosmetic outcome are scarce, 2 related studies did not show compromised cosmetic satisfaction with CS [25,31].

Notably, multiple tumors and technical factors could influence the determination of margin status and these are generally beyond the control of surgeons. DCIS was a critical issue that deserved special attention owing to its tendency to be multifocal [49]. Margin-positive patients with DCIS had a worse prognosis compared with margin-positive patients with invasive ductal carcinoma [52]. NAC treatment might lead to tumor shrinkage in a mosaic or honeycomb pattern [53]. Consequently, surgical resection of the tumor mass in NAC-treated patients tends to leave more residual carcinoma in the cavity compared with resection in non-NAC-treated patients [49]. Chen et al. showed that the cavity margin status was significantly associated with locoregional recurrence in NAC-treated patients but not in non-NAC-treated patients [49]. In addition, tumor size [29,40,45], tumor grade [29,46,49], vascular invasion [29,46,49], and lymph node metastasis [15,29,40,45,49] have been suggested to be correlated with the cavity-shave margin status. These factors should be carefully considered when planning the extent of the cavity shave margin [29].

This meta-analysis has several limitations. Because retrospective studies or non-randomized studies were included [14,37,48], recall bias and selection bias were unavoidable. The definition of the positive cavity shave margin was not consistent, and the standard of positivity varied between studies with respect to the distance from the cut edge. Although the guideline defines no ink on tumor as an adequate margin [10], the inking method might lead to false-positive results owing to shrinkage of the specimen, ink seeping into the specimen, or dislocation of malignant cells towards the margins [36]. This criteria was recently criticized to result in increased positive CSM compared with the standard ≥2 mm margin in lumpectomy [54–56]. The thickness of the cavity shave and whether or not it was oriented were at the discretion of the surgeon [15]. In addition, it was rather difficult to standardize the excised volume of CSM [25]. The majority of studies did not perform intraoperative frozen section analysis of the shaved cavity margin [37]. Several confounding factors, such as post-BCS radiotherapy [57], have been suggested to be closely associated with future recurrence or survival. Lumpectomy specimens with narrower margins were more likely to have residual disease in two or more SCM [54]. In addition, the advancement of intraoperative imaging techniques could help eliminate re-excisions [58,59].

## Conclusion

In conclusion, our meta-analysis confirmed that additional CS was effective in reducing margin involvement and the reoperation rate when compared with partial mastectomy alone. CS did not seem to be associated with an excessive excised tissue volume or poor cosmetic outcomes. Although the included studies suffered from high heterogeneity, they represented the best evidence currently available. Reassuringly, our findings were consistent with the results from the largest single-center RCT on the cavity shave margin.

## Supporting Information

S1 File Search strategy used for PubMed database. (DOCX)

S2 File PRISMA 2009 Checklist. (DOC)

S1 Table Quality assessment of randomized controlled trials by the Jadad scale. (DOCX)

S2 Table Quality assessment of non-randomized studies by the modified Newcastle-Ottawa scale (NOS). (DOCX)
