# Supplementary material for: Cavity Shaving plus Lumpectomy versus Lumpectomy Alone for Patients with Breast Cancer Undergoing Breast-Conserving Surgery: A Systematic Review and Meta-Analysis
Source: PLoS One. 2017 Jan 3;12(1):e0168705. doi: 10.1371/journal.pone.0168705 (PMC5207394; doi:10.1371/journal.pone.0168705)
Supplement: S1 File — (DOCX) [file pone.0168705.s001.docx]

**Keywords used for PubMed database.**

#1. Cavity

#2. Shave

#3. Shaving

#4. Margin

#5. #2 OR #3 OR #4

#6. #1 AND #5

#7. shave margin

#8. #6 OR #7

#9. Breast

#10. Cancer

#11. Tumor

#12. Neoplasm

#13. Carcinoma

#14. #10 OR #11 OR #12 OR #13

#15 #9 and #14

#16. breast-conserving

#17. partial mastectomy

#18. Lumpectomy

#19. #15 OR #16 OR #17 OR #18

#20. #8 AND #19
