# Supplementary material for: Cavity Shaving plus Lumpectomy versus Lumpectomy Alone for Patients with Breast Cancer Undergoing Breast-Conserving Surgery: A Systematic Review and Meta-Analysis
Source: PLoS One. 2017 Jan 3;12(1):e0168705. doi: 10.1371/journal.pone.0168705 (PMC5207394; doi:10.1371/journal.pone.0168705)
Supplement: S1 Table — (DOCX) [file pone.0168705.s003.docx]

**Supplemental Table 1. Quality assessment of randomized controlled trials by the Jadad scale.**

| Author (year) | Randomization | | Blinding | | Description of withdrawals and dropouts | Total score |
| --- | --- | --- | --- | --- | --- | --- |
|  | Mentioned | Appropriate | Metioned | Appropriate |  |  |
| Chagpar et al. (2015) | 1 | 1 | 1 | 1 | 1 | 5 |
| Jone et al. (2016) | 1 | 1 | 1 | 1 | 1 | 5 |
