# Supplementary material for: Cavity Shaving plus Lumpectomy versus Lumpectomy Alone for Patients with Breast Cancer Undergoing Breast-Conserving Surgery: A Systematic Review and Meta-Analysis
Source: PLoS One. 2017 Jan 3;12(1):e0168705. doi: 10.1371/journal.pone.0168705 (PMC5207394; doi:10.1371/journal.pone.0168705)
Supplement: S2 Table — (DOCX) [file pone.0168705.s004.docx]

**Supplemental Table 2. Quality assessment of non-randomized studies by the modified Newcastle-Ottawa scale (NOS).**

| Author (year) | Adequate definition  of cases with breast cancer | Representativeness  of cases undergoing cavity shaving | Ascertainment  of cavity shave margins | Outcomes of interest were not present before cavity shave | Control of confounding factors | Assessment  of outcomes | Adequate follow-up (> 1 year) | Total score |
| --- | --- | --- | --- | --- | --- | --- | --- | --- |
| Macmillan et al. (1994) | 1 | 1 | 1 | 1 | 1 | 1 | 1 | 7 |
| Keskeka et al (2004) | 1 | 1 | 1 | 1 | 1 | 1 | 1 | 7 |
| Camp et al. (2005) | 1 | 1 | 1 | 1 | 1 | 1 | 1 | 7 |
| Cao et al. (2005) | 1 | 1 | 1 | 1 | 1 | 0 | 0 | 5 |
| Janes et al. (2006) | 1 | 0 | 1 | 1 | 1 | 1 | 0 | 5 |
| Huston et al. (2006) | 1 | 1 | 1 | 1 | 1 | 1 | 0 | 6 |
| Jacobson (2008) | 1 | 1 | 1 | 1 | 1 | 0 | 0 | 5 |
| Marudanayagam et al. (2008) | 1 | 1 | 1 | 1 | 0 | 1 | 0 | 5 |
| Povoski et al. (2009) | 1 | 0 | 1 | 1 | 1 | 1 | 0 | 5 |
| Lovrics et al. (2009) | 1 | 1 | 1 | 1 | 1 | 1 | 0 | 6 |
| Tengher-Barna et al. (2009) | 1 | 1 | 1 | 1 | 1 | 1 | 0 | 6 |
| Rizzo et al. (2010) | 1 | 1 | 1 | 1 | 1 | 1 | 0 | 6 |
| Zavagno et al. (2010) | 1 | 1 | 1 | 1 | 1 | 1 | 0 | 6 |
| Coopey et al. (2011) | 1 | 1 | 1 | 1 | 1 | 1 | 1 | 7 |
| Feron et al. (2011) | 1 | 0 | 1 | 1 | 1 | 1 | 0 | 5 |
| Hequet et al. (2011) | 1 | 1 | 1 | 1 | 1 | 1 | 0 | 6 |
| Kobbermann et al. (2011) | 1 | 1 | 1 | 1 | 1 | 1 | 0 | 6 |
| Wolf et al. (2011) | 1 | 0 | 1 | 1 | 1 | 1 | 0 | 5 |
| Mook et al. (2012) | 1 | 1 | 1 | 1 | 1 | 1 | 0 | 6 |
| Unzeitig et al. (2012) | 1 | 1 | 1 | 1 | 1 | 1 | 0 | 6 |
| Yang et al. (2012) | 1 | 1 | 1 | 1 | 1 | 1 | 0 | 6 |
| Hequet et al. (2013) | 1 | 1 | 1 | 1 | 1 | 1 | 1 | 7 |
| Bolger et al. (2015) | 1 | 0 | 1 | 1 | 1 | 1 | 0 | 5 |
| Pata et al. (2016) | 1 | 1 | 1 | 1 | 1 | 1 | 1 | 7 |
